# Strengthening lung cancer screening in Europe: fostering participation, improving outcomes, and addressing health inequalities through collaborative initiatives in the SOLACE consortium

**DOI:** 10.1186/s13244-024-01814-5

**Published:** 2024-10-22

**Authors:** Hans-Ulrich Kauczor, Oyunbileg von Stackelberg, Emily Nischwitz, Joanna Chorostowska-Wynimko, Monika Hierath, Coline Mathonier, Helmut Prosch, Pamela Zolda, Marie-Pierre Revel, Ildikó Horváth, Martina Koziar Vašáková, Pippa Powell, Miroslav Samarzija, Torsten Gerriet Blum, and SOLACE Consortium

**Affiliations:** 1 Department of Diagnostic and Interventional Radiology, Heidelberg University Hospital, Translational Lung Research Center (TLRC), German Center for Lung Research (DZL), Heidelberg, Germany; 2 Department of Genetics and Clinical Immunology, National Institute of Tuberculosis and Lung Diseases, Warsaw, Poland; 3 European Institute for Biomedical Imaging Research, Vienna, Austria; 4 Department of Biomedical Imaging and Image-Guided Therapy, Medical University of Vienna, Vienna, Austria; 5 Department of Radiology, Hôpital Cochin, AP-HP, Paris, France; 6 Faculté de Médecine, Université Paris Cité, Paris, France; 7 National Koranyi Institute for Pulmonology, Budapest, Hungary; 8 Department of Pulmonology, University of Debrecen, Debrecen, Hungary; 9 Department of Respiratory Medicine, Thomayer University Hospital, Prague, Czech Republic; 10 Lungs Europe, Brussels, Belgium; 11 School of Medicine, University of Zagreb, Zagreb, Croatia; 12 Department for Respiratory Diseases Jordanovac, University Hospital Centre Zagreb, Zagreb, Croatia; 13 Medical School Berlin, Berlin, Germany; 14 Department of Pneumology, Lungenklinik Heckeshorn, Helios Klinikum Emil von Behring, Berlin, Germany

**Keywords:** SOLACE, Early detection of cancer, Lung, Low-dose computed tomography

## Abstract

**Abstract:**

The Strengthening the Screening of Lung Cancer in Europe (SOLACE) initiative, supported by Europe’s Beating Cancer Plan, is dedicated to advancing lung cancer screening. This initiative brings together the most extensive pan-European network of respiratory and radiology experts, involving 37 partners from 15 countries. SOLACE aims to enhance equitable access to lung cancer screening by developing targeted recruitment strategies for underrepresented and high-risk populations. Through comprehensive work packages, SOLACE integrates scientific research, pilot studies, and sustainability efforts to bolster regional and national screening efforts across EU member states.

**Critical relevance statement:**

The SOLACE project aims to facilitate the optimization and implementation of equitable lung cancer screening programs across the heterogeneous healthcare landscape in EU member states.

**Key Points:**

- The effectiveness of lung cancer screening is supported by both scientific evidence and now increasing legislative support.
- SOLACE aims to develop, test, and disseminate tools to facilitate the realization of lung cancer screening at both a national and regional level.
- Previously underrepresented populations in lung cancer screening will be targeted by tailored recruitment strategies.
- SOLACE forms the first pan-European network of experts poised to drive real-world implementation of lung cancer screening.

## Introduction

Lung cancer has been the leading cause of cancer deaths in both sexes in EU member states, since 2017 [1, 2]. It is the second and third most commonly diagnosed cancer among males and females in EU member states, respectively. The incidence of lung cancer was 97.2 per 100,000 for males and 43.9 per 100,000 for females, according to recent estimates [3]. The potential for early detection at a curative stage with low-dose computed tomography (LDCT) screening is well documented, including at least a 20% reduction in mortality among high-risk individuals [4–6]. Over the past two decades, compelling evidence supporting LDCT screening stems from numerous randomized clinical trials conducted in both the USA and Europe. The two largest trials, the National Lung Screening Trial (NLST) in the USA and the Dutch-Belgian Randomized Lung Cancer Screening Trial (NELSON), collectively involved more than 69,000 participants. Specifically, in Europe, robust evidence has been generated from significant studies in the Netherlands (NELSON), Italy (MILD/BioMILD, ITALUNG, and DANTE), France (DEPISCAN), Germany (LUSI), Denmark (DLCST), and the United Kingdom (UKLS) [6–14]. Despite the substantial evidence provided by these landmark studies, and ongoing discussions about the introduction of lung cancer screening programs (LCSP), the national implementation of such programs in Europe has been slow. To date, only three out of the 27 EU member states, Croatia, Czech Republic, and Poland, have established national LCSP.

The respiratory and radiology community has been continuously advocating for the wider implementation of low-dose CT as the most effective tool for early and reliable detection of lung cancer [15]. This position has been strongly supported by the European Society of Radiology (ESR) and European Respiratory Society (ERS) as expressed in the ERS/ESR joint statement [16], as well as by the European Society of Thoracic Imaging (ESTI), European Society of Thoracic Surgeons, and European Society for Radiotherapy and Oncology to name a few. The SAPEA (Scientific Advice for Policy by European Academies) report confirmed the robust scientific foundation supporting the rollout of lung cancer screening in Europe in March of 2022. In September of 2022, the European Commission put forth a recommendation advocating for step-wise implementation of lung cancer screening across the EU. Following extensive deliberations, the EU Council of Ministers for Health not only adopted the proposal but also approved funding at both national and European levels in December of 2022. As part of the flagship initiative of Europe’s Beating Cancer Plan, lung, prostate, and gastric cancer screening was incorporated into the EU cancer screening strategy [16]. Subsequently, three programs—strengthening the screening of lung cancer in Europe (SOLACE), Prostate Cancer Awareness and Initiative for Screening in the European Union, [17], and Toward Gastric Cancer Screening Implementation in the European Union—received funding from the EU4Health program with the explicit goal of facilitating the implementation of these screenings. The expectation of the simultaneous initiation of the implementation of lung, prostate, and gastric cancer screening is to foster collaboration and alignment and to identify potential synergies among these screening programs.

**Fig. 1:**
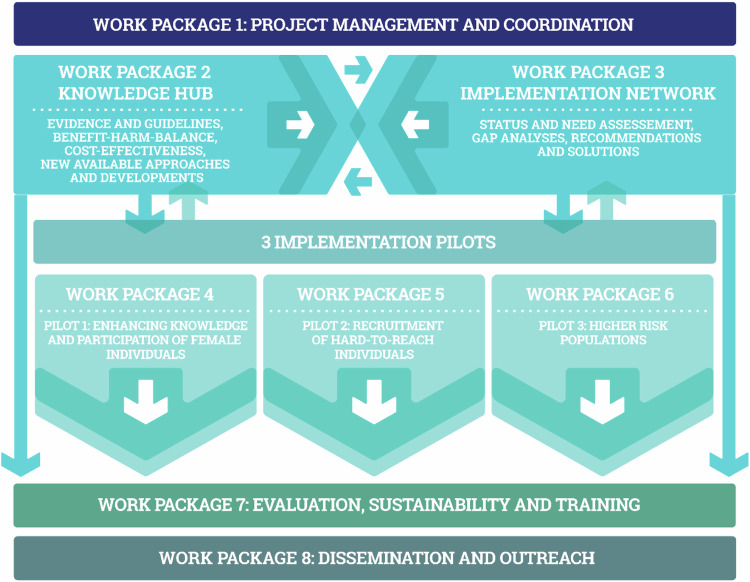
Overview of SOLACE work packages

The SOLACE project, initiated in April 2023 and to conclude in March 2026, embodies a pivotal effort to optimize and usher in the implementation of cutting-edge LCSP across EU member states. This joint initiative led by the ESR and ERS acts as a natural continuation of the past efforts and implementation of the ERS/ESR joint statement [16]. The project consortium steering this initiative comprised of 37 partners, including academic hospitals, health authorities, research institutes, associations, and organizations representing patients and professionals. These representatives cover a spectrum of 15 EU member states, highlighting a diverse and cooperative scientific approach, collaborating in eight work packages (Fig. 1). Work Package 1 oversees overarching project coordination and management. Work Package 2 focuses on evidence-based implementation and universal lung cancer screening guidelines, considering benefit and harm balance, cost-effectiveness, and epidemiology of the overall population at risk. Work Package 3 focuses on establishing the current state of play of lung cancer screening in EU member states through the formation of National Expert Teams. Work Packages 4–6 include implementation pilots targeting previously underrepresented groups in lung cancer screening. The guideline and implementation package (GIP) will rely on the gap analysis performed by WP3, as well as the real-world data from WP4-6. Work Package 7 evaluates ongoing progress and ensures the long-term viability of the project. The establishment of the European Lung Cancer Screening Alliance (ELSCA) will ensure continued impact beyond the project duration and sustained efforts of the stimulation of standardized lung cancer screening training programs across disciplines. Work Package 8 ensures effective communication and dissemination of the project to all relevant stakeholders at each stage. All eight Work Packages rely on frequent exchange and synergistic action, which is achieved through inter-work packages and consortia-level exchange.

**Fig. 2:**
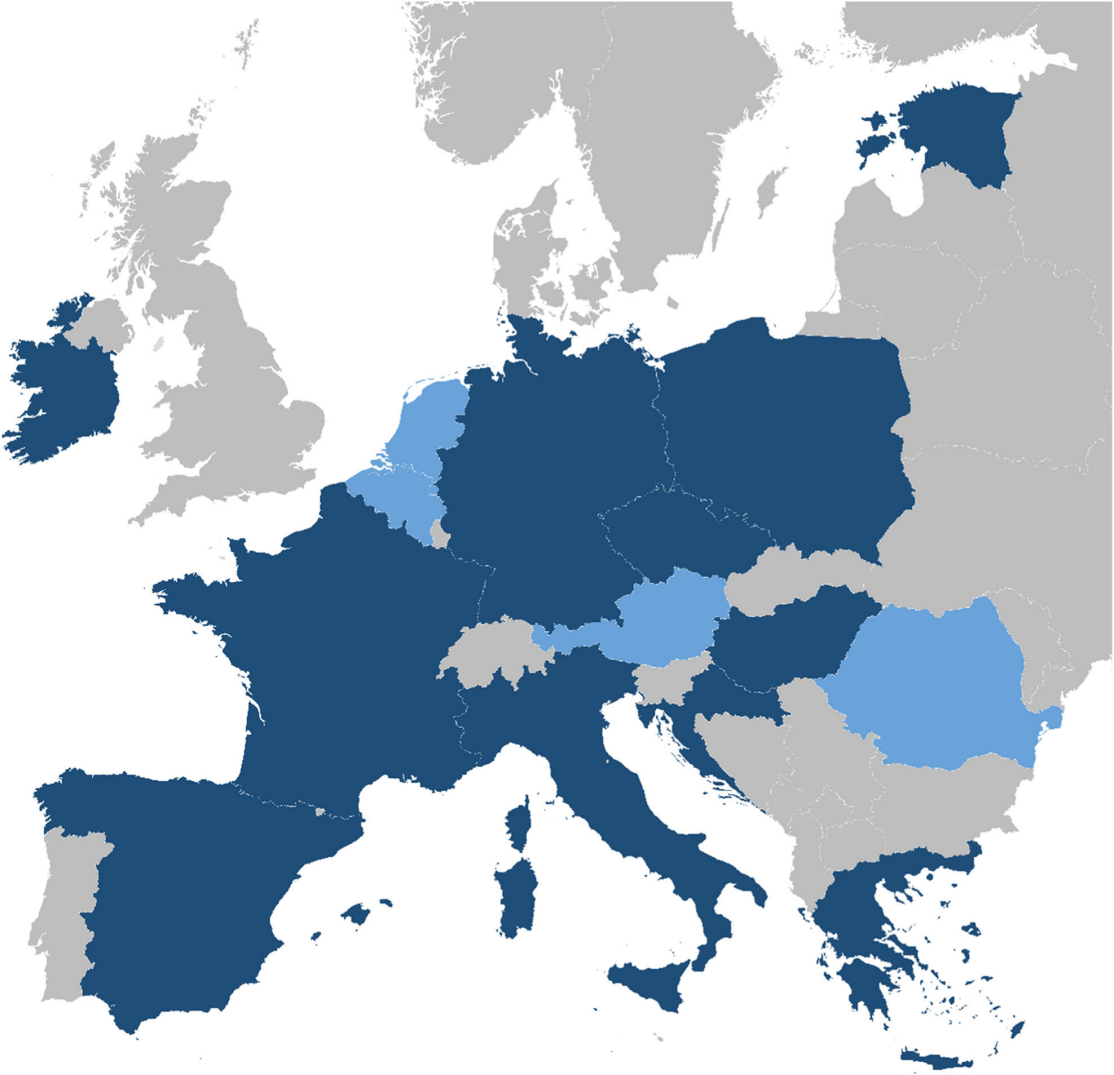
Overview of pilots. All SOLACE partners are indicated in blue. Dark and light blue represent the countries that do and do not have ongoing pilots and/or programs involved in SOLACE, respectively. Light gray countries are not involved in SOLACE

SOLACE marks the first comprehensive pan-European lung cancer screening network linking all relevant experts, making this an ideal environment for the development of these resources (Fig. 2).

## GIP and knowledge hub

The extensive network of interdisciplinary experts within SOLACE will create a comprehensive guideline, implementation, and quality assurance package (GIP). This will include a compilation of pathway modules, evidence-based guidelines, and technical and methodology papers accessible to all who wish to design, plan, pilot, and roll out an LCSP. The GIP methodology will follow the grades of recommendation, assessment, development, and evaluation approach and most importantly has been aligned with the EU Joint Research Centre Cancer Screening Guideline Group.

As part of the GIP, key factors such as the epidemiology of populations at risk, benefit-harm balance, and cost-effectiveness will be considered at each step in the pathway. To cause the least harm and ensure the highest degree of cost-effectiveness, it is critical to evaluate and target the appropriate high-risk groups. Major harms such as radiation exposure, false positive findings, and complications of diagnostic workups are being critically considered. To systematically evaluate cost-effectiveness, SOLACE health economics experts will apply existing cost-effectiveness models, fine-tuning these models to reflect the heterogeneous landscape of healthcare systems across Europe.

To host the documents developed and compiled within this project, as well as the data and results gained from the analysis of prior trials and future pilots, the SOLACE Knowledge Hub has been established. The SOLACE website (www.solacelung.eu), which is a part of the knowledge hub, will serve as a dissemination platform for SOLACE's “Guidelines, quality assurance, and implementation package” (GIP). Moreover, the open access will contain consistent consortium progress updates and other informational documents. The SOLACE Database is established to collect comprehensive clinical data of LCS participants from ongoing pilots.

## Evaluation of state of play of lung cancer screening in EU member states and EEA countries

While lung cancer screening pathways using LDCT may seem straightforward initially, the complexity comes in the details, and it becomes clear that the controlled conditions of published trials from the USA and Western Europe cannot be applied as a single “one size fits all” blueprint. Therefore, Work Package 3 aims to create dedicated reports on the state of play of lung cancer screening in the 27 EU member states and three EEA countries, which are being assessed in both the in-depth surveys and semi-structured interviews. The semi-structured interviews will allow the flexibility of gathering general and country-specific information from all relevant national experts. This will allow a comprehensive evaluation of the current implementation of lung cancer screening in EU countries. Templates will be prepared to assess the implementation and quality of programs within the European member states, in collaboration with the prostate and gastric cancer consortia. This will uniquely position the SOLACE consortium to evaluate the needs and gaps that serve as roadblocks to the national implementation of lung cancer screening.

## Implementation pilots to identify and enroll distinctive populations currently underrepresented in LCSP

Given the heterogeneous landscape of healthcare systems, SOLACE partner experts from ongoing national screening programs and pilot studies and trials will contribute insights and internally available screening data. As part of Work Packages 4, 5, and 6, each of these sites are targeting thus far previously underrepresented populations in lung cancer screening including: women, hard-to-reach individuals, and individuals with previously identified co-morbidities with a propensity to lung cancer. While each program employs unique recruitment methods, SOLACE has created core recruitment material representing best practices for sites to adapt to their needs. Posters and flyers have been created to target women, with specific messages concerning sex-specific risk of lung cancer. Additionally, many sites are combining efforts with local breast cancer screening programs. Four hard-to-reach populations are being targeted: ethnic minorities, participants with social deprivation, participants who are located remotely from the screening center, and participants recruited via occupational medicine. Individuals who are ethnic minorities and/or socially deprived, are being recruited by an ambassador program in which members from the community are trained in the importance of lung cancer screening and how to disseminate that message. To target individuals who are located remotely, mobile CT truck units are being placed in strategic locations to allow individuals to more easily access screening sites. Pulmonologists and radiologists are being approached to encourage their patients with significant co-morbidities to seek lung cancer screening. Patients with chronic obstructive pulmonary disease and fibrotic interstitial lung disease (fILD), as well as cancer survivors and patients who have undergone organ transplantation, are being specifically targeted. fILD patients present a unique challenge in that the early radiologic signs of cancer are often missed within the fibrotic changes. Further research of effective screening measures, as well as diagnosis and treatment of fILD patients is an unmet clinical need.

All LCSPs target high-risk individuals, based on their age, smoking history, and various co-morbidities. Each program has unique sets of inclusion and exclusion criteria to fulfill the specific regional and national needs (Table 1). Amongst these sites are the three national screening programs in Europe (Croatia, Czechia, and Poland), as well as various pilot and implementation programs [18, 19]. In Estonia and Croatia, a general practitioner recruitment-based program is ongoing and is planned to occur in Ireland [20]. For both running programs, the recruitment via general practitioner practices has been highly effective with thus far 4963 and 30,174 baseline screened participants in Estonia and Croatia, respectively. In France, the Lung Cancer Screening in French women using low-dose CT and Artificial Intelligence for DEtection (CASCADE) project is ongoing, which is the first LCSP to only recruit female participants [21]. The mobile-CT unit-based program “Lyon Mobile Screening and Prevention Solution for Lung Cancer in AuRA Region” (MobILYAD) is set to begin in late 2024. In Germany, the HANSE-SOLACE study using mobile-CT units targeting the northern German region, as well as the EU-funded “4-IN THE LUNG RUN” trials are ongoing [22]. The Greece Hellenic Ministry of Health is partnering with SOLACE to begin its first lung cancer screening with sites in both Athens and Crete. After the two Hungarian multi-center nationwide pilots (HUNCHEST and HUNCHEST 2), HUNCHEST-SOLACE is now ongoing with an intense focus on hard-to-reach individuals [23, 24]. In Ireland, the Lung Health Project trial is set to begin screening in late 2024, and in addition to targeting individuals through GPs, they will support access to remote communities via a mobile CT unit. Additionally, the Rete Italiana screening polmonare (RISP) program is participating in SOLACE, with additional retrospective data contributions from two previous Italian randomized-controlled clinical trials, MILD/BioMILD (Multicentric Italian Lung Detection) and PEOPLHE (model for optimized implementation of early lung cancer detection: prospective evaluation of preventive lung health) [25–28]. Finally, in Spain, the International Early Lung Cancer Action Program (I-ELCAP) supported sites in Pamplona and Madrid are contributing both retrospective data to SOLACE. The invaluable knowledge, experience, and data that will be gained and shared in these studies will be essential in the next steps towards the implementation of lung cancer screening across Europe.

**Table 1:** Program SOLACE data contributing center with unique sets of inclusion and exclusion criteria to fulfill the specific regional and national needs

			Inclusion criteria				Exclusion criteria	
Program SOLACE data contributing center	Type	Start year	Age	Pack years	Quit smoking since	PLCO score	Last CT scan	Previous lung cancer
Croatia Croatian National LCS Program (CNLCSP)—UHCZ Screening Program Website	Ongoing National Program	2020	50–75	≥ 30 PY	≤ 15 years		≤ 1 year	≤ 5 years
Czech Republic Czech Early Detection Program for Lung Cancer (CZ-LCS)—TN Screening Program Website	Ongoing National Program	2022	55–74	≥ 20 PY	–	–	–	–
Estonia Estonian Pilot Study of LCS (EE-LCS)—UTRATU Screening Program Website	Pilot trial	2022	55–74	≥ 20 PY	< 15 years	≥ 1.51%	≤ 1 year	≤ 5 years
France LCS in French women using LDCT and AI for Detection (CASCADE)—APHP Screening Program Website	Randomized controlled trial	2022	50–74	≥ 20 PY	≤ 15 years	–	≤ 2 year	Excluded
France MobILYAD—HCL via CT-truck	Pilot trial	2024	50–75	≥ 20 PY	≤ 15 years	–	≤ 1 year	Excluded
Germany Towards INdividually tailored INvitations, screening INtervals, and INtegrated co-morbidity reducing strategies in LCS (4-ITLR)—DKFZ Screening Program Website	Randomized controlled trial	2022	55–77	≥ 30 PY	≤ 15 years	≥ 1.83%	≤ 1 year	Excluded
Germany HANSE—SOLACE-MHH, GHD, and UKSH via CT-truck Screening Program Website	Pilot trial	2023	55–79	–	–	≥ 1.58%	≤ 1 year	≤ 5 years
Greece Greece-SOLACE (EL-LCS)—MoHGR	Pilot trial	2024	50–75	≥ 20 PY	≤ 15 years	–	–	Excluded
Hungary Hungarian LDCT Lung Cancer Screening Program-SOLACE (HUNCHEST-SOLACE)—NKIP, OEKP/PDC, OR/SZSZBVK	National LCSP	2023	50–75	≥ 20 PY	≤ 15 years	–	≤ 1 year	≤ 5 years
Ireland Lung Health Check (LHC)—RCSI Screening Program Website	Randomized controlled trial	2024	55–74	–	–	≥ 1.51%	≤ 1 year	≤ 5 years
Italy RISP—UNIPR, INT-IRCCS Screening Program Website	National LCSP	2022	55–75	≥ 30 PY	≤ 15 years	–	≤ 1 year	≤ 5 years
Poland Ogólpolski Program Wczesnego Wykrywania Raka Płuca (WWRP)—IGICHP Screening Program Website	National LCSP	2020	50–74	≥ 20 PY	≤ 15 years	–	–	–
Spain International Early Lung Screening Action Program (I-ELCAP)—UNAV Screening Program Website	Pilot trial	2001	≥ 40	≥ 10 PY	≤ 10 years	–	–	≤ 5 years

*LCS* lung cancer screening, *PY* pack year, *PLCO* prostate, lung, colorectal, and ovarian score

## Training and sustainability

The resources and knowledge gained during the course of the SOLACE project will be established to allow long-lasting impact in Work Package 7. As part of this initiative, SOLACE’s goal is to stimulate the creation of dedicated training programs for various disciplines involved in screening. As the technical aspects of lung cancer screening require specific knowledge and expertise there is a great need for standardized training. An example of such a training initiative is the Lung Cancer Screening Certification Project by the ESTI, which aims to ensure that lung cancer screening is performed at a quality level comparable to the trials that have demonstrated its effectiveness (https://www.myesti.org/lungcancerscreeningcertificationproject/). Similarly, coordinated training programs in other disciplines will not only accelerate the implementation of lung cancer screening across Europe but also maintain the high quality of these programs.

Throughout SOLACE, Work Package 8 ensures that the key findings and messages from SOLACE are being disseminated to influence the future of lung cancer screening. The positive momentum of SOLACE will be transformed into a sustainable movement by the creation of the European Lung Cancer Screening Alliance led by Work Package 7 in close conjunction with Work Package 8. This alliance will link the expertise and experiences of all relevant stakeholder groups at the EU and national levels. The resulting transnational network structures and knowledge exchange will facilitate and accelerate the creation of national LDCT LCSP and empower more EU member states to start and accomplish this complex implementation process in their countries.

## Conclusion and perspectives

The SOLACE project is poised to transform lung cancer screening throughout EU member states by harnessing the expertise of the largest pan-European network of respiratory and radiology experts to date. SOLACE utilizes a multidisciplinary and comprehensive approach, encompassing evidence-based guidelines, pilot programs, and robust dissemination and sustainability strategies. It sets the stage for the effective and equitable implementation of lung cancer screening across EU member states.

## Abbreviations

CASCADE: Lung cancer screening in French women using low-dose CT and artificial intelligence for detection
ERS: European Respiratory Society
ESR: European Society of Radiology
ESTI: European Society of Thoracic Imaging
fILD: Fibrotic interstitial lung disease
GIP: Guideline and implementation package
LCSP: Lung cancer screening programme
LDCT: Low-dose computed tomography
MILD/BioMILD: Multicentric Italian lung detection
MobILYAD: Lyon mobile screening and prevention solution for lung cancer in AuRA region
NELSON: Dutch-Belgian randomized lung cancer screening trial
PEOPLHE: Prospective evaluation of preventive lung health
RISP: Rete Italiana screening polmonare
SOLACE: Strengthening the screening of lung cancer in Europe
